# KRAS in Colorectal Cancer: Tumorigenesis, Surgical Implications and Evolving Treatment Target

**DOI:** 10.3390/curroncol33090514

**Published:** 2026-08-28

**Authors:** Sahar Iftikhar, Alexander H. Xiao, Zhaohui Jin, Emad H. Aly

**Affiliations:** 1 School of Medicine, Medical Sciences, and Nutrition, University of Aberdeen, Aberdeen AB25 2ZD, UK; u07si23@abdn.ac.uk; 2 Department of Medical Oncology, Mayo Clinic, Rochester, MN 55905, USA; 3 Aberdeen Royal Infirmary, Aberdeen AB25 2ZN, UK

**Keywords:** colorectal cancer, KRAS, G12C, tumourigenesis, targeted therapy

## Abstract

Purpose: This review aims to provide an updated overview of Kirsten rat sarcoma viral oncogene homologue (KRAS) mutations in colorectal carcinoma (CRC), focusing on their role in tumorigenesis, prognostic implications, and recent advances in targeted therapy. Major findings: KRAS mutations occur in approximately 40% of colorectal cancers and play a central role in tumour initiation and progression through constitutive activation of MAPK pathways. Clinically, KRAS mutations are well established as predictors of resistance to anti-EGFR therapy. Increasing evidence also supports their role as prognostic biomarkers, with KRAS-mutant tumours associated with increased recurrence risk and reduced survival, including in patients undergoing hepatic metastasectomy. Therapeutically, recent advances, most notably KRAS G12C inhibitors and combination strategies targeting upstream or parallel pathways, have expanded treatment options, although efficacy varies across KRAS mutation subtypes. Conclusions: KRAS mutations have important implications for the behaviour, prognosis, and management of colorectal cancer. Integrating KRAS mutational status into clinical decision-making may enable more personalised prognostication and treatment strategies. Continued research is required to broaden effective targeted therapies for the diverse spectrum of KRAS-mutant disease.

## Simple Summary

Mutations in the Kirsten rat sarcoma viral oncogene homologue (KRAS) play an important role in colorectal cancer and are linked to higher morbidity and mortality rates while also being historically difficult to treat. This review summarises the pathophysiology which leads the mutation to tumour formation, its impact on disease prognosis, and an up-to-date overview of the drug classes which target the mutation, ranging from the approved use of specific variants to the broader drugs still in preclinical development stages.

## 1. Introduction

Colorectal carcinoma (CRC) represents a major global health challenge with it being the third-most diagnosed cancer and the second leading cause of cancer-related mortality worldwide [1]. Over 1.9 million new CRC cases and approximately 935,000 deaths were reported in 2020 alone [2]. CRC rates vary by region, with higher incidence rates observed in high-human-developmental-index (HDI) regions, likely influenced by lifestyle factors such as diet and obesity. However, incidence rates are rapidly increasing in low-HDI regions, reflecting ongoing changes in dietary habits and urbanisation [3]. The incidence of early-onset colorectal cancer (eoCRC, defined as age at diagnosis < 50) has been rising in the past several decades in 27 of 50 countries while rates of CRC in older adults (age >= 50) are stabilising or declining in some countries including the United States [4].

While environmental and lifestyle factors, including high consumption of red and processed meats, low fibre intake, smoking, obesity, sedentary lifestyle, and alcohol use, contribute significantly to CRC risk [5], these external factors alone do not fully explain individual susceptibility to the disease. The interaction between exposome and inherited genetic predispositions play a critical role in determining CRC risk [3].

Early research on CRC focused on tumour morphology and clinical staging; however, subsequent molecular studies have found that a series of defined genetic events drive the development of the disease [6,7]. Over time, our understanding of these genetic alterations has expanded—from the identification of inherited conditions like familial adenomatous polyposis (FAP) and Lynch syndrome (hereditary non-polyposis colorectal cancer, HNPCC), to the characterisation of somatic mutations in key oncogenes and tumour suppressors. Among these, at least three genetic mutations are typically required for CRC to develop, with frequent involvement of APC, TP53, and KRAS [8,9]. A concise overview of the most common hereditary syndromes and somatic mutations in CRC with clinical implications are summarised in Table 1, providing context for the central focus of this Kirsten rat sarcoma viral oncogene homologue (KRAS) review.

Among these alterations, KRAS mutations are particularly prevalent and have been widely studied due to their involvement in colorectal, lung, and pancreatic cancers. Their significance lies not only in its frequency, but also in its functional impact on tumour behaviour, including its aggressiveness and response to targeted therapies [13]. Its role in CRC development, from early adenoma formation to malignant transformation, alongside its impact on therapeutic resistance, remains a priority in cancer research. These insights pave the way toward improved prognostic tools and novel targeted therapies that could ultimately enhance patient outcomes.

## 2. Methods

Two databases were used for all searches performed: PubMed Central and OVID Embase. A filter for articles published between January 2010 and June 2026 was applied for the search terms “colorectal cancer” AND “KRAS” AND “targeted therapy.” However, important seminal articles or groundbreaking work before the search time frame were included. Approximately 100 relevant articles were identified during the initial search. They were then screened by title, abstract, and full text to ensure they met the following criteria: human research on CRC involving KRAS mutations, published in English with full text availability, and presenting original data (clinical trials, cohort studies, and translational research) or systematic reviews. Non-human studies, editorials, and conference abstracts were excluded.

The selected literature was assessed and narratively reviewed. Quality and risk of bias were considered qualitatively, with priority given to high-impact clinical trials and recent research.

## 3. KRAS in CRC

**Figure 1:**
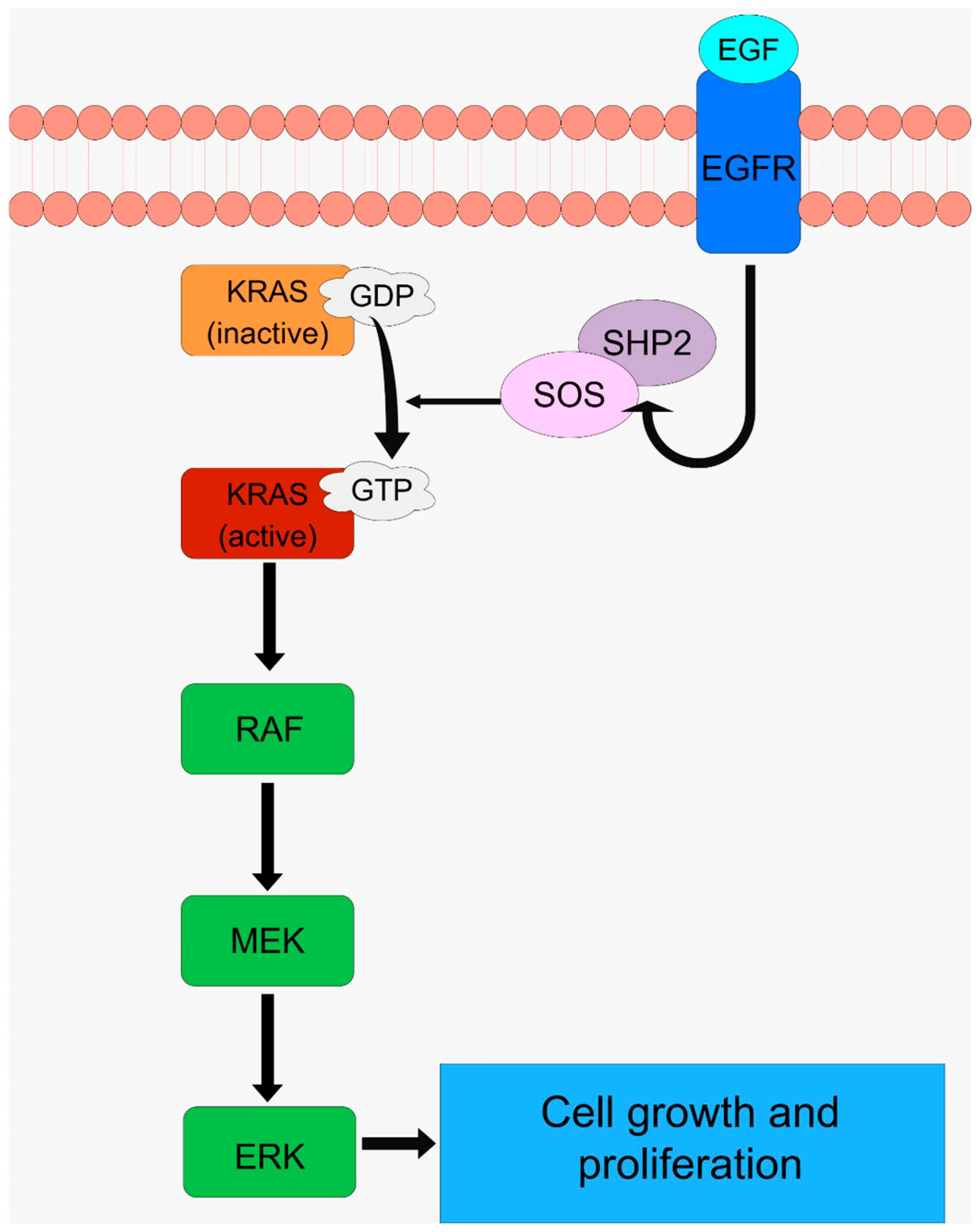
Diagram of the KRAS-RAF-MAPK signalling pathway. Ligand binding to EGFR activates adaptor proteins (SHP2, SOS) that stimulate KRAS to initiate MAPK pathway. This figure was created using draw.io (diagrams.net).

The KRAS gene belongs to the rat sarcoma (RAS) family of proto-oncogenes. These genes encode for RAS proteins which can alternate between active and nonactive states when bound to guanosine triphosphate (GTP) and guanosine diphosphate (GDP) molecules, respectively. The protein exists in a balance between its GTP-bound and GDP-bound states, which allows it to turn on and off the intracellular signalling of growth factor receptors, such as the epidermal growth factor receptor (EGFR) and other tyrosine kinase receptors [14]. When active, RAS proteins can trigger a number of transduction cascades, particularly the mitogen-activated protein kinase pathway (MAPK) [15,16]. Figure 1 illustrates how EGFR activation, via SOS and SHP2, leads to KRAS activation and RAF-MEK-ERK signalling, therefore promoting cell life proliferation and survival.

Mutations in KRAS impair the protein’s intrinsic GTPase activity, causing inefficient hydrolysis of GTP. This defect keeps KRAS in an active, GTP-bound state, which in turn drives persistent stimulation of downstream pathways that promote cellular survival [17].

KRAS mutations represent the predominant mutation among RAS-family alterations, accounting for roughly 86% of RAS-driven cancers [18]. In CRC, they are present in about 40% of cases, making it one of the most common genetic alterations in the disease and are more frequent among right- than left-sided colon cancers [19]. The mutations typically occur at codons 12 and 13, where specific variants have distinct biological behaviours with potential prognostic and therapeutic implications [13,20].

CRC harbouring oncogenic KRAS mutations (KRAS-mutant CRC) are generally associated with a poorer prognosis than KRAS wild-type CRC, which lacks oncogenic mutations. Mutations at codon 12, such as G12V and G12D, have worse survival outcomes and higher recurrence rates. Among all subtypes, G12C appears to confer the most unfavourable prognosis [21,22]. Co-mutational burden is also important, as combined alterations in other pathways, such as with TP53, can portend even more aggressive disease patterns [23].

Given its prevalence and prognostic impact, KRAS plays a pivotal role in the development of CRC, acting as an important driver in both the traditional adenoma–carcinoma sequence and, to a lesser extent, the serrated neoplasia pathway. Understanding how KRAS contributes to these distinct routes of tumorigenesis offers a valuable insight into its functional impact on CRC progression.

## 4. CRC Pathways

### 4.1. Adenoma–Carcinoma Sequence

The first hypothesis regarding the tumorigenesis of colorectal tumours was the Fearon–Vogelstein model. This model described tumour formation through a series of genetic mutations which ultimately activate oncogenes and inactivate tumour suppressor genes [24]. The most notable pathway following this model involves the loss of the tumour suppressor genes APC and TP53, as well as the addition of the KRAS oncogene [25]. The series begins with a mutation in the APC gene in the Wnt signalling pathway that helps regulate intestinal cell proliferation and homeostasis. In normal circumstances, APC forms part of a protein complex associated with the degradation of the main mediator of the pathway (β-catenin); this process helps keep Wnt signalling under control. However, mutations in APC disrupt the pathway regulation, resulting in β-catenin accumulation and therefore hyperactivation of Wnt signalling. This leads to uncontrolled cell proliferation and adenomatous polyp formation [26,27,28].

**Figure 2:**
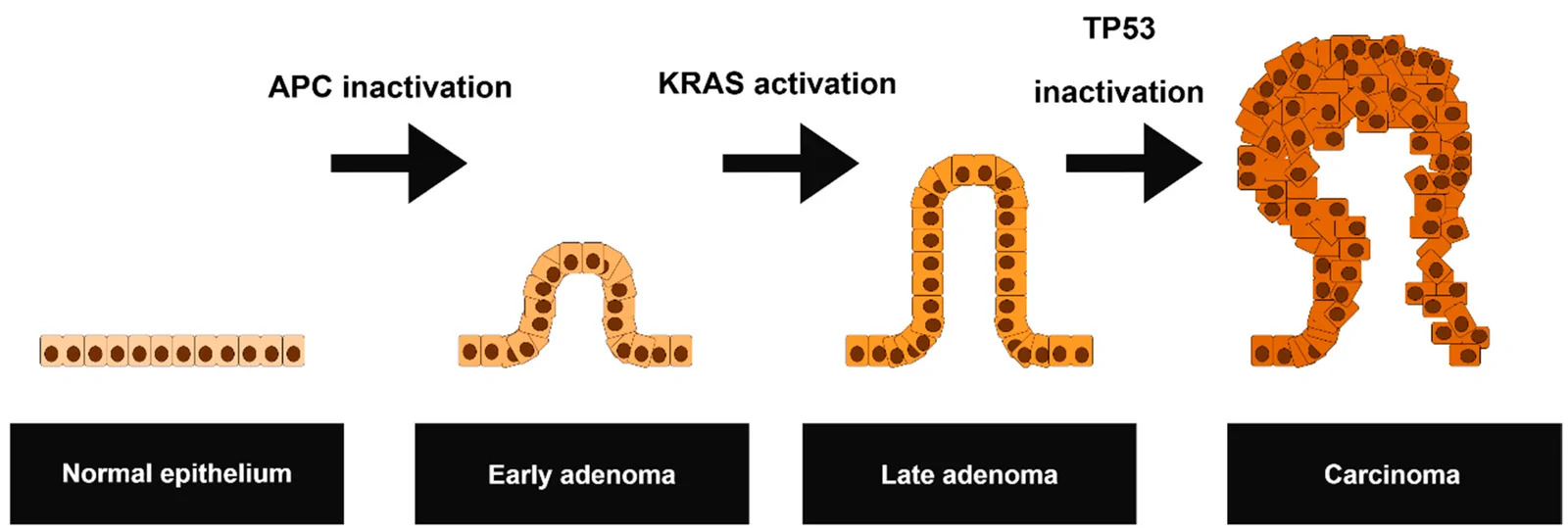
Location of the KRAS mutation in the adenoma–carcinoma pathway. This figure was created using draw.io (diagrams.net).

In the classic adenoma–carcinoma sequence of CRC, APC inactivation typically occurs first, followed by KRAS activation, with TP53 inactivation almost always occurring as the final event (Figure 2) [25]. While KRAS mutations do not directly cause malignancy, they facilitate progression of early-stage adenomas to a later stage by sustaining proliferative signalling and preventing apoptosis (see KRAS in CRC). These mutations impair the KRAS GTPase activity, leading to persistent activation of downstream pathways and tumour growth [15,16].

TP53 mutations are generally considered a late event in the sequence, frequently arising in high-grade adenomas and associated with the transition to invasive carcinoma [25,29]. Most cancers, and up to 60% of CRC patients, exhibit TP53 mutations, which correlate with a poorer prognosis [30,31]. The p53 transcription factor, encoded by TP53, is important to the cell cycle as it prevents tumorigenesis through the induction of DNA repair or apoptosis in response to damaged or irreparable cells [32]. In CRC, most of the mutations are missense, leading to a dysfunctional p53 protein that not only loses its tumour-suppressive mechanisms, but can also exert gain-of-function effects, enhancing genomic instability which further accelerates accumulation of genetic alterations that promote metastasis [30,33,34].

### 4.2. Serrated Pathway

**Figure 3:**
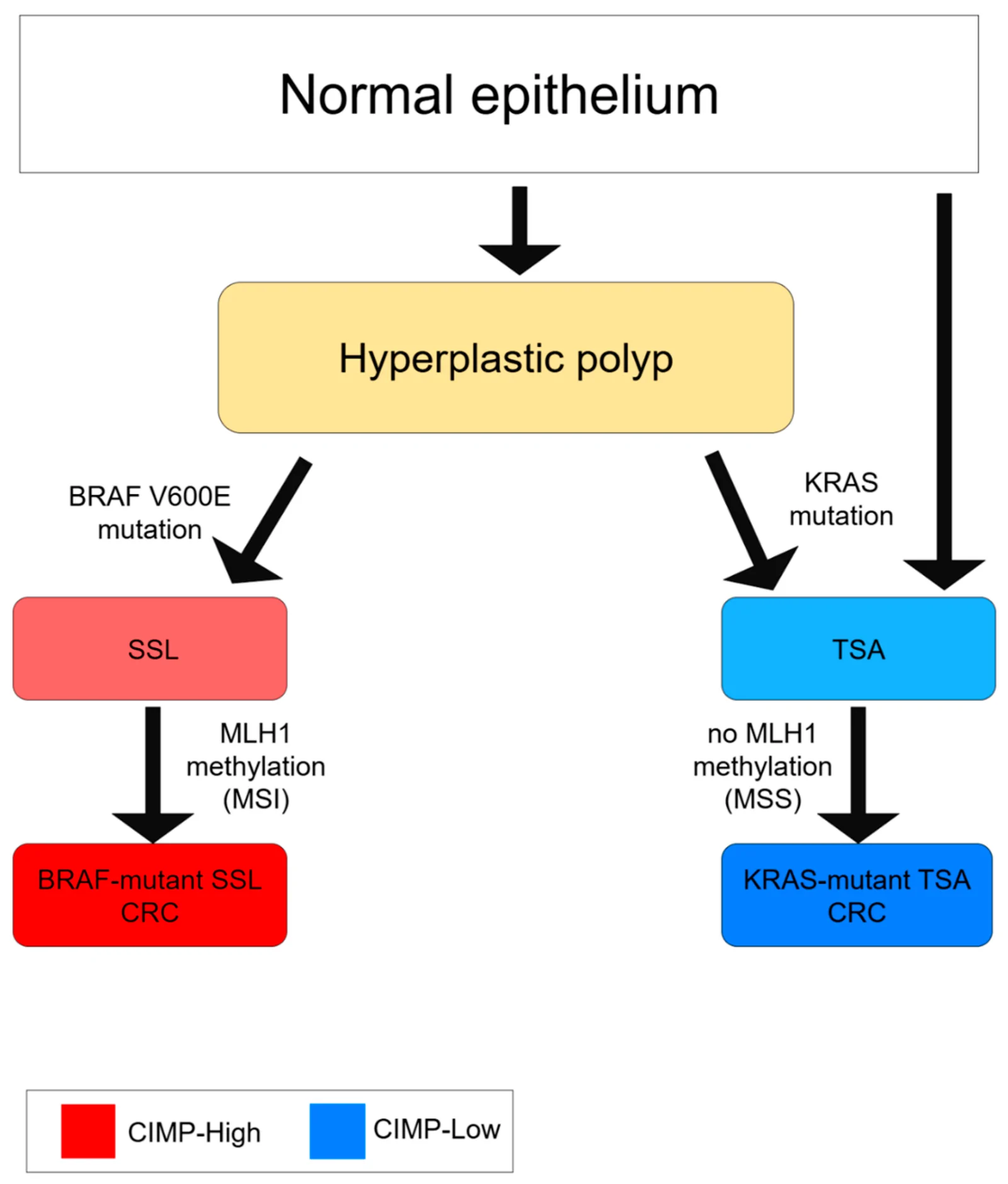
Flow chart showing the sessile and traditional serrated pathway. This figure was created using draw.io (diagrams.net).

While the adenoma–carcinoma series accounts for most CRC cases, up to 30% develop via an alternative serrated pathway. This pathway begins with serrated polyps, named for their characteristic saw-tooth appearance [35]. These polyps can develop into two main malignant precursors: sessile serrated lesions (SSLs) and traditional serrated adenomas (TSAs). SSLs, the more common lesion in this pathway, are strongly linked to the BRAF V600E mutation, whereas TSAs are typically associated with KRAS mutations. BRAF mutations can occur in TSAs but are less common [36,37]. Both lesions can arise from hyperplastic polyps, but TSAs may also develop de novo in certain cases [38]. The mutational background tends to influence progression; hyperplastic polyps with BRAF mutations generally evolve into SSLs, whereas those with KRAS mutations are more likely to give rise to TSAs (Figure 3). Within SSLs, mutations in KRAS/BRAF are more frequently in the right side of the colon and commonly present with microsatellite instability [19].

SSLs may advance to carcinoma when the MLH1 gene becomes heavily methylated, which often leads to DNA changes called microsatellite instability (MSI). However, some SSL cancers remain microsatellite stable (MSS), meaning they do not show these changes. In contrast, cancers that arise from TSAs are usually MSS [39,40].

Regardless of microsatellite status, both tumour types typically display a CpG island methylator phenotype (CIMP), in which hypermethylation silences tumour suppressor genes [41,42]. CIMP can be classified as high or low, depending on the extent of methylation. SSL cancers are usually high, whereas TSA cancers are low [40]. Rarely, MSI CRCs may also carry KRAS mutations [43].

These alternative tumorigenic routes underscore the genetic heterogeneity of CRC and highlight the recurring role of KRAS across distinct molecular contexts. Given its frequent occurrence and functional significance, KRAS stands out as a pivotal driver in CRC pathogenesis.

## 5. Clinical Implications

### 5.1. Response to Anti-EGFR Therapy

RAS mutations influence both prognosis and treatment selection and therefore should be considered when evaluating treatment options. One of its most important implications is that it predicts a poorer response to anti-EGFR therapies. Anti-EGFR monotherapy has little place in the treatment of KRAS mutations as the proteins are able to completely bypass EGFR inhibition by activating MAPK pathways, and thereby promoting tumour survival despite anti-EGFR therapy. For this reason, RAS-mutant metastatic CRC patients tend to be treated with cytotoxic chemotherapy and anti-VEGF agents. This is different to the treatment of RAS wild-type metastatic CRC as the treatment choice is often determined by which side the tumour is present. Malignancies on the left side in RAS wild-type CRC are more likely to receive cetuximab or panitumumab alongside chemotherapy agents FOLFOX or FOLFIRI. Right-sided tumours more commonly have co-occurring BRAF V600E mutations and are treated using chemotherapy and anti-VEGF therapy. It is important to note that MMR/MSI status heavily impact management options for KRAS mutations. For example, immunotherapy is the first-line therapy option for MSI-H/dMMR metastatic CRC, regardless of RAS mutation status [44].

Testing for specific KRAS mutations is now standard practice to stratify patients for anti-EGFR therapy eligibility, specifically KRAS and NRAS exons 2, 3, and 4, examining codons 12, 13, 59, 61, 117 and 146 as mutations in these exons predict lack of benefit from conventional anti-EGFR monoclonal antibodies [45]. In practice, broad next-generation sequencing is preferred to simultaneously identify other actionable or trial-relevant mutations and MMR/MSI status. As KRAS mutations typically emerge early in carcinogenesis, its status is highly concordant between the primary tumour and metastatic sites, allowing for either site to be used in testing [45].

Approximately 40–50% develop acquired resistance within 4–8 months of treatment initiation [46,47]. This resistance tends to arise from mutations in the extracellular portion of EGFR or by the constitutive activation of pathways driving proliferation independent of EGFR. Moreover, secondary KRAS mutations can emerge during treatment as well as mutations in BRAF, MET or HER2 amplification and PI3K pathway activation [46,47]. Tumours with acquired RAS mutations have been found to behave more aggressively than those with EGFR-mediated resistance and are frequently associated with shorter PFS and more limited tumour shrinkage [48,49,50]. Recent work has also identified non-genomic resistance to EGFR therapy, via transformation to Paneth-like cells via increased SMAD1-FGFR3 signalling [51]. These findings support molecular reassessment at progression.

### 5.2. Liquid Biopsy

The emergence of resistance underscores the importance of early detection of evolving mutations, which can guide treatment changes. Liquid biopsy has emerged as a promising approach for this purpose, providing a non-invasive method for analysing tumour-derived material from blood or other bodily fluids [52]. One of the key components of liquid biopsy is the analysis of circulating tumour DNA (ctDNA), which consists of fragmented tumour-derived DNA that are released into the circulation through tumour cell apoptosis and necrosis [53]. While tissue biopsy remains the gold standard for molecular diagnosis, liquid biopsy serves as a valuable complementary tool for identifying RAS and BRAF mutations and for longitudinal monitoring during therapy. Its clinical use has been demonstrated not only in CRC but also other malignancies, including breast and non-small-cell lung cancer (NSCLC) [52]. In particular, anti-EGFR rechallenge has emerged as a strategy for select patients by selecting for patients found to have a decline in KRAS mutant resistant clones. In the CHRONOS trial, ctDNA was utilised to select patients with metastatic CRC for panitumumab rechallenge based on absence of resistance mutations in RAS, BRAF and EGFR. Disease control rate was 63% in the selected cohort. A 2026 meta-analysis by Kuznetsova et al. of three phase II trials with metastatic CRC undergoing anti-EGFR rechallenge found that rechallenge improved disease control rate, objective response rate and progression-free survival. However, an overall survival benefit was not observed, suggesting that rechallenge may be most suitable for patients in whom tumour shrinkage or short-term disease control is critical [54].

By allowing assessment of tumour-derived genetic material, liquid biopsy can be used to detect CRC at earlier stages, facilitating prompt intervention. The integration of liquid biopsies into clinical practice is valuable for both early KRAS mutation detection, ongoing monitoring of resistance during anti-EGFR therapy and guiding anti-EGFR rechallenge [55]. However, ctDNA is not without its limitations. Earlier-stage and low volume disease may shed minimal ctDNA, thus impacting sensitivity. This may further be impacted by site of disease, such as reduced sensitivity with peritoneal or lung metastasis [53]. Furthermore, clinical implementation itself may ultimately be limited by turnaround time as well as cost and reimbursement. Additionally, KRAS status can evolve over treatment, with reports of “regression” in which a detectable KRAS-mutant clone disappears [56]. This is thought to be driven by clonal selection rather than genetic reversion and thus should not be used as a marker of disease control or to determine surgical candidacy. KRAS mutational regression has particularly been described in oligometastatic disease and may have implications in supporting longitudinal molecular monitoring and consideration of treatment re-sensitisation.

### 5.3. Prognostic and Surgical Implications of KRAS Targeting

Beyond their established role in predicting resistance to anti-EGFR therapy, KRAS mutations have emerged as an important prognostic biomarker in CRC. Increasing evidence suggests that KRAS-mutant status provides independent prognostic information, tending to be more aggressive, which translates into higher rates of recurrence and poorer survival outcomes, even in patients undergoing curative surgery [13,57]. This has significant implications for surgical decision-making and postoperative management.

In this context, detection of KRAS-mutant ctDNA has demonstrated substantial prognostic value. Preoperative identification of KRAS-mutant ctDNA has been shown to be independently associated with an increased risk of postoperative recurrence following curative CRC resection, likely reflecting the presence of minimal residual disease not detectable by conventional imaging modalities [45,53,58,59]. As such, preoperative KRAS-mutant ctDNA testing may help identify patients at higher risk of early recurrence who could benefit from more stringent postoperative surveillance or adjuvant therapy.

The prognostic impact of KRAS mutations is also evident in the metastatic setting, particularly among patients undergoing liver resection for CRC liver metastases. Molecular analysis of resected metastases has demonstrated that RAS mutations, including KRAS, are associated with significantly worse overall survival following hepatic surgical resection as well as increased risk for extrahepatic recurrence [60]. Notably, this adverse effect appears to be more pronounced in patients with eoCRC, with particularly poor outcomes observed in patients before the age of 40 [61]. This age-dependent effect supports the idea that KRAS-mutant tumours arising in younger individuals may represent a biologically distinct and more aggressive disease subset. Interestingly, several recent reports have also found that patients with polymetastatic disease are more enriched for KRAS mutations compared with those with oligometastatic disease. This suggests a genomic distinction between these two groups, rather than oligometastatic disease simply representing lower volume disease [62,63].

Collectively, this data suggests that KRAS mutational status has the potential to inform surgical decision-making in CRC. Ultimately, KRAS mutations are a risk modifier rather than an absolute contraindication to curative or repeat surgery. Their presence does not warrant more extensive surgery but could identify patients at increased risk of recurrence despite complete resection. In such cases, KRAS status, especially when combined with preoperative ctDNA analysis, may support the use of neoadjuvant or perioperative systemic therapy, influence the timing of surgery, or justify more intensive postoperative surveillance strategies.

### 5.4. Challenges in KRAS Targeting

Due to the relevance of KRAS mutations on disease prognosis and treatment selection, researchers have explored the concept of targeting KRAS directly. However, studies have confirmed the protein to have limited numbers of available binding sites due to its small structure and smooth surface. Additionally, KRAS naturally exhibits high affinity for GTP, which exists at great concentrations. KRAS structures can also differ significantly between variants, thus limiting therapeutic efficacy across multiple variants [64]. These factors collectively hinder the development of effective competitive inhibitors [65]. Even with an effective inhibitor, bypassing mutations or feedback activation loops can ultimately restore signalling.

Thus, research efforts have focused on combination therapies to target KRAS indirectly, including agents which can chemically alter its expression, disrupt its membrane localisation, and interfere with its interaction with downstream mediators. Unfortunately, most of these approaches have been unable to achieve complete suppression of KRAS-driven tumour formation [66]. Table 2 summarises the challenges of designing KRAS inhibitors and how those difficulties have been handled.

## 6. Emerging Therapies

### 6.1. G12C Inhibitors

The development of a new drug class targeting the specific KRAS G12C mutant variant has marked a turning point in the management of CRC. Despite only making up ~4% of all CRC cases, KRAS G12C has demonstrated to be associated with a higher mortality compared to other variants [67].

KRAS G12C inhibitors work by binding to the switch-II pocket on the G12C mutant and initiating a conformational change that will lock the protein in an inactivated state [68]. Sotorasib and Adagrasib are the most promising agents of this class, with ongoing trials proving their effectiveness against tumours of the G12C variant. Both drugs share commonly reported side effects being diarrhoea, nausea and vomiting, and fatigue [67,69].

One limitation of G12C inhibitor monotherapy is its susceptibility to adaptive resistance due to feedback activation of upstream EGFR signalling. KRAS G12C inhibitors were initially developed in non-small-cell lung cancer where monotherapy was found to be efficacious. However, CRC retains strong EGFR-driven feedback signalling; this compensatory pathway reactivation restores downstream MAPK signalling despite KRAS inhibition, thereby limiting the efficacy of G12C inhibitors when used alone [45]. This has prompted investigation into combination regimens with anti-EGFR agents such as cetuximab and panitumumab, which have shown to be effective in treatment of metastatic CRC [67,70]. To date, sotorasib plus panitumumab as well as adagrasib plus cetuximab are approved options for adults with KRAS G12C-mutated metastatic CRC after prior fluoropyrimidine, oxaliplatin, and irinotecan-based chemotherapy [71].

Certain studies have found that a combination therapy using SHP2 and SOS1 inhibitors may strengthen the antitumour effects of G12C inhibitors and anti-EGFR therapy combination. Both SHP2 and SOS1 inhibitors have complementary mechanisms of action by restricting different mediators in the RAS signalling cascade, which results in inhibition of pathway reactivation and augmentation of G12C activity [72].

### 6.2. Other KRAS Variants

In addition to G12C, other KRAS mutations such as G12D, G12V, and G13D represent a larger proportion of CRC cases but currently lack approved direct inhibitors. These variants have posed significant challenges due to the absence of the switch-II binding pocket to which covalent G12C inhibitors may bind, making drug design more complex. Nevertheless, emerging efforts are focused on developing new therapeutic strategies to directly target these prevalent mutations. Evidence has shown that the novel agent HRS-4642 functions as an effective KRAS G12D inhibitor in the treatment of NSCLC [73]. However, being relatively new with limited clinical data in CRC, HRS-4642 is not currently approved for the treatment of G12D-mutant CRC. More recently, additional KRAS G12D-targeted therapies have emerged in pancreatic ductal adenocarcinoma (PDAC). INCB161734 and ASP3082 have been evaluated in PDAC, demonstrating antitumour efficacy as monotherapy and in combination with other chemotherapy regimens such as modified FOLFIRINOX. While these agents are presently being investigated in PDAC, their activity against KRAS G12D highlights their potential relevance for future therapeutic use in G12D-mutant CRC [74,75].

KRAS G12V-selective inhibitors are currently in development, with preclinical trials exploring the tumour regressing effects of EFTX-G12V [76]. EFTX-G12V is an EGFR-directed G12V inhibitor which works by restricting the protein expression via direct suppression of KRAS G12V mRNA [77]. There are other agents with potential to target this variant still in early stages of development. Of these are tri-complex inhibitors, RM-048 and VRTX-171, which are currently being studied to establish their efficacy against the G12V variant [78,79].

Despite the general consensus that RAS-mutant tumours are not significantly impacted by anti-EGFR drugs, rising evidence suggests that the KRAS G13D mutation may retain some amount of sensitivity to these agents. Although the underlying physiology is not entirely understood, some researchers have suggested that the inadequate binding of KRAS G13D to the tumour suppressor NF1 resultantly impairs its function of inhibiting wild-type RAS isoforms. Under this circumstance, an EGFR blockade would therefore decrease the concentration of GTP-bound RAS molecules and stop the signalling cascade [80]. Further research has been undertaken in this field to explore methods that may enhance this sensitivity. One particular approach is by using honokiol, a natural biphenolic compound, which can refine the effectiveness of cetuximab by preventing autophagy and RAS activation [81]. Novel compound YK1 is another method that acts as a small molecule inhibitor to target the HER2-ELF3-KRAS transcriptional axis which otherwise exists in high concentrations in KRAS G13D malignancies. By interfering with this series, HER2 and KRAS become heavily downregulated, and therefore lead to an increase in cetuximab sensitivity [82]. Although compounds increasing anti-EGFR therapy sensitivity require further clinical trials, these findings suggest promising approaches for targeting KRAS G13D variants. Table 3 summarises the main KRAS mutations and their implications in treatment options.

### 6.3. RAS(ON) Inhibitors

As the use of KRAS inhibitors has found its way into clinical practice, RAS has been identified as a practical target in CRC. RAS(ON) inhibitors have emerged as a broader drug class that can directly inhibit active GTP-bound RAS proteins [83]. KRAS G12C inhibitors can only target the GDP-bound states of one specific variant, meanwhile RAS(ON) inhibitors act more generally to hinder active signalling across multiple RAS isoforms, including KRAS, NRAS, and HRAS. These inhibitors have a wider scope of mutational targets, and can even extend to specific variants [84]. Daraxonrasib (RMC-6236) is a first-in-class, pan-RAS(ON) inhibitor that has been demonstrated to have promising activity against common RAS mutations, including KRAS G12D, G12V, G12A, G12S, G13D, and Q61X variants [84,85,86].

Daraxonrasib binds intracellulary to cyclophilin A; this binary complex then engages the GTP-bound RAS, forming a tri-complex and blocking RAS-effector binding, thereby suppressing downstream signalling [84]. Resistance to daraxonrasib is primarily driven by impairment of daraxonrasib binding, commonly through secondary RAS Y64 mutations, or enhancement of native RAS-RAF signalling, making RAF harder for daraxonrasib to displace, typically through RAS Y71 mutations or kinase-dead/hypoactive BRAF mutations [87]. To date, the strongest evidence has been recorded in PDAC where it has shown promising antitumour activity and is being evaluated in multiple late-stage clinical trials [86,88]. Daraxonrasib is also being investigated in other solid tumours, such as CRC and NSCLC, where ongoing studies are evaluating its efficacy both as a monotherapy and standard chemotherapy regimens [89,90]. Table 4 demonstrates how information of KRAS biology can be utilised by CRC surgeons to enhance management decisions for patients.

## 7. Targeting Vertical and Parallel Pathways

New strategies to block KRAS pathways are increasingly focused on shutting down both vertical and parallel signalling routes to overcome resistance. Vertical inhibition targets compounds directly above or below KRAS in the same pathway [91]. One key example is SHP2, a protein that helps stimulate KRAS and maintain its active state for longer. Several drugs designed to block SHP2, such as SHP099, TNO155, and RMC-4630, are now being tested in early studies [92,93]. Another set of drugs block SOS1, a similar KRAS activator, with agents like BI-1701963 and BI-3406 in development [94]. Other approaches aim to disrupt KRAS connections further downstream, such as Emicoron, which reduces KRAS production by binding to specific DNA structures called G-quadruplexes [95].

Parallel pathway inhibition takes a different route by targeting signals outside the main KRAS pathway that malignant cells use to survive when KRAS is blocked. Onvansertib is a promising drug in this category. It has shown its antitumour activity by inhibiting PLK1 proteins that normally regulate the cell cycle [96]. Moreover, Onvansertib provides promising results when combined with standard chemotherapy, producing tumour shrinkage in about one-quarter of patients with KRAS-mutant colorectal cancer in early trials. Larger studies are now testing this drug as part of a first-line treatment [97]. Together, these strategies highlight a shift toward targeting multiple points in cancer signalling rather than KRAS alone.

## 8. Future Directions

There are ongoing developments within KRAS-directed therapy that show promise. Many KRAS-mutant tumours have an immune-suppressive microenvironment through methods including reduction in T-cell recruitment and promotion of tumour-associated-macrophages. Immunological approaches have thus been investigated, such as engineered T-cell receptors or vaccines targeting KRAS neoantigens although overall efficacy remains uncertain [98]. Tumour-infiltrating lymphocyte (TIL) production has been investigated as well in small studies, including one in which a KRAS G12D reactive TIL resulted in objective regression in one patient with metastatic colorectal cancer [99]. Immunotherapy, while utilised in microsatellite instability-high or mismatch repair-deficient colorectal cancer, remains investigational as a combination therapy in KRAS-mutant disease. Other emerging therapies include proteolysis-targeting chimaeras, which are being investigated and may provide greater efficacy by inducing degradation of KRAS, rather than simple KRAS occupancy [100]. Future prospective studies could benefit from investigating combined modality KRAS treatment. Additionally, trials further investigating utility of ctDNA to guide perioperative therapy, surveillance or repeat ablation or surgery are warranted.

## 9. Conclusions

Genetic mutations play an extensive role in CRC aetiology. Of all the main mutational statuses, KRAS mutations stand out as having a vast influence on the disease. Studies into the genetic mutation have not only found its direct involvement in the adenoma–carcinoma sequence of CRC tumorigenesis, but have also shown it to be a likely factor for poorer prognosis and anti-EGFR resistance, which ultimately impacts treatment selection for patients with the mutation. Although KRAS has historically proven to be a difficult target to develop competitive inhibitors against, novel compounds have emerged in recent years to target specific variants, such as KRAS G12C inhibitors which have demonstrated high efficacy and antitumour effects. Broader drug classes like RAS(ON) inhibitors, proteolysis-targeting chimaeras and immunologic strategies are currently in development to cover a wider range of mutations in the RAS family. Further research on KRAS genetic mutations and clinical trials are required to develop effective therapies for a wider population in patients with KRAS-mutant CRC.

## Figures and Tables

**Table 1:** Selected genetic alterations in colorectal cancer. Data adapted [10,11,12].

Category	Key Genetic Alteration	Genes Involved	Key Features	Prevalence in CRC
Germline (inherited cancer predisposition syndrome)	Lynch syndrome (HNPCC)	MSH2, MLH1, MSH6, PMS2	Autosomal dominant; MMR defect; MSI-H	~3%
	Familial adenomatous polyposis (FAP)	APC	Autosomal dominant. Up to thousands of adenomas; ~100% risk of CRC	<1%
Somatic (tumour-acquired mutation)	APC mutation	APC	Persistent Wnt pathway activation	70–80%
	KRAS mutation	KRAS	Persistent MAPK signalling; MSS	~40%
	BRAF V600E mutation	BRAF	Persistent MAPK signalling	~10%

Abbreviations: HNPCC, hereditary non-polyposis colorectal cancer; MMR, mismatch repair; MSI-H, microsatellite instability (high); MSS, microsatellite stable; MAPK, mitogen-activated protein kinase.

**Table 2:** Challenges and strategies in KRAS targeting.

Challenge in KRAS Targeting	Strategies to Overcome
Few binding sites accessible	Previously unrecognised sites have been identified, such as the switch-II pocket
High affinity of KRAS for GTP/GDP	Targeting allosteric pockets rather than the GTP/GDP binding site to avoid competition
Different KRAS structure between variants	Development of allele selective agents
Compensatory feedback activation	Combination therapy of KRAS inhibitor with anti-EGFR therapy
Development of bypassing mutations	Combination therapy with other directed agents guided by molecular profiling

**Table 3:** Summary of current treatment status of KRAS mutation subtypes.

KRAS Mutation and Prevalence	Prognostic Implications	Current and Emerging Therapies
G12C (~4%)	Most consistently associated with adverse prognosis depending on stage and co-mutations	Adagrasib plus cetuximab and sotorasib plus panitumumab are approved; SHP2/SOS1 combinations are investigational
G12D (~12–15%)	Common and generally intermediate, metastatic behaviour is heterogenous	No direct inhibitor approved; potential benefit from HRS-4642, INCB161734 and ASP3082
G12V (~9%)	Adverse, second to G12C	No direct inhibitor approved; KRAS G12V-selective EGFR-directed inhibitors in development
G13D (~7%)	Prognostic implications are inconsistent	No direct inhibitor approved; investigational interest in anti-EGFR and combination therapy

**Table 4:** Key take home points regarding KRAS in colorectal cancer.

Decision Point	Role of KRAS
Selection of systemic therapy	Molecular profiling including extended RAS testing should be obtained as it identifies anti-EGFR resistance, targeted treatment opportunities and clinical trial eligibility.
Surgical candidacy	KRAS-mutant disease supports a more aggressive biology and thus should be taken into account with metastatic burden, disease tempo and response to systemic therapy in selecting patients for surgery.
Resectable liver metastasis	KRAS mutations confer increased risk of recurrence but are not a contraindication to surgery and should not be used to determine extent of surgery.
Postoperative surveillance and residual disease assessment	KRAS-mutant ctDNA can help identify patients at increased risk of recurrence and support closer surveillance intervals. However, false negatives must be taken into account, especially in low-volume disease.
Disease recurrence or progression	KRAS mutation status may evolve during treatment and thus repeat molecular testing can help identify development of and potential for alternate therapy for resistant clones.

## Data Availability

This manuscript does not report data generation or analysis.
